# The effects of PCB126 on intra-hepatic mechanisms associated with non alcoholic fatty liver disease

**DOI:** 10.1186/s40200-015-0218-2

**Published:** 2015-12-10

**Authors:** Marie-Pier Boucher, Caroline Lefebvre, Natalie Ann Chapados

**Affiliations:** 1 Institut de recherche de l`Hôpital Montfort, Hôpital Montfort, 713 Montreal Road, Ottawa, ON K1K 0T2 Canada; 2 School of Human Kinetics, Faculty of Health Sciences, University of Ottawa, Ottawa, ON Canada; 3 La Cite, Ottawa, ON Canada

**Keywords:** Steatosis, Environmental contaminants, Liver, Rats

## Abstract

**Background:**

Non alcoholic fatty liver disease (NAFLD) results from alteration in lipid synthesis and elimination mechanisms such as very-low density lipoprotein (VLDL) production and *de novo* lipogenesis. Persistent organic pollutants (POPs) are chemicals that were mostly used historically as pesticides, solvents, flame retardant, and other applications. Among POPs, polychlorinated biphenyls (PCB) have been recognized to be of environmental and potential toxicologic concerns. Specifically, PCB126 could act as endocrine disruptors and has recently been associated with hepatic fat accumulation. The purpose of the study was to investigate the effects of PCB126 on the molecular development of NAFLD using hepatocyte and rat models.

**Methods:**

Hepatocytes were exposed to PCB 126 for 72 h and lipid accumulation in cells was quantified by Oil-Red-O. Rats were injected with a single dose of PCB126 or vehicle. Seven days later, liver triglycerides (TAG) content was measured along with protein quantification of hepatic microsomal triglyceride transfer protein (MTP), sterol regulatory element-binding protein 1c (SREBP1c) and diacylglycerol O-acyltransferase 2 (DGAT-2).

**Results:**

Exposure to PCB126 resulted in significant increases of lipid accumulation in hepatocytes (38 %, *P* <0.05) and hepatic TAG concentrations (64 %, *P* <0.001) in rats compared to respective control groups. Rats with fatty livers depicted lower MTP (40 %, *P* <0.02), higher SREBP1c (27 %, *P* < 0.05) and DGAT-2 (120 %, *P* < 0.02) protein content levels compared to Placebo group in rats.

**Conclusions:**

It seems that exposure to PCB126 has an important emerging role in the pathophysiology of NAFLD by 1) altering elimination mechanisms such as VLDL synthesis and secretion, through MTP; and 2) increasing hepatic TAG synthesis mechanisms through DGAT 2 and SREBP1c.

## Background

Liver plays an essential role in the biogenesis of major metabolites such as lipids. However, an important increased in hepatic fat depot leads to the development of NAFLD. NAFLD is the most prevalent liver disease in North America and reaching approximately 20 % of the population worldwide, and affecting both adults and children [1, 2]. NAFLD has become a common cause of chronic metabolic diseases such as steatohepatitis, diabetes [3] and metabolic syndrome [4]. Historically NAFLD was linked to overnutrition, physical inactivity and pharmacology. However, emerging studies have demonstrated that additional contributing factors may play a role in the development of NAFLD, such as persistent organic pollutants (POPs).

Persistent organic pollutants are chemicals that were mostly used historically as pesticides, solvents, and flame-retardants and have been released in the environment. In North America, POPs have been recognized to be of environmental and potential toxicologic concern, as a result, a variety of POPs have been banned from use since the late 1970’s [5]. However, due to their persistence and lipophilicity, these compounds will remain present in the environment for decades and accumulate in living organisms [6]. POPs also accumulate in adipose tissue and are continuously released from adipose tissue to the circulation and to vital organs with lipid content [7]. In humans, a National Health and Nutrition Examination Survey (NHANES) study showed that the total participants had detectable circulating POPs levels [8]. POPs regroup polychlorinated biphenyls (PCBs), a family of 209 different congeners. A total of 1.3 million tons of PCBs were manufactured prior 1977 for use in electrical and other industrial applications [9]. PCB126 is the most potent and ubiquitous in the environment amongst congeners [10]. Recent studies have reported that the presence of PCB126 could act as endocrine disruptors at the liver level and lead to fatty liver [11].

Hepatic lipid accumulation results from altered intrahepatic TAG elimination and synthesis mechanisms. Specific key-molecules such as MTP is involved in lipid elimination pathways through VLDL production and secretion, while synthesis pathways regroup DGAT-2 and SREBP1c.

Hepatic VLDL production is a complex process involving several enzymes to carry TAG from liver to peripheral tissues [12]. MTP plays a pivotal role in the assembly and secretion of apoB-containing lipoproteins [13]. MTP is a heterodimeric protein expressed on the luminal side of ER composed of a catalytic subunit of 97 kDa and a multifunctional protein of 55 kDa, disulfide isomerase [14]. Molecular processes such as fatty acid synthesis and esterification also regulate hepatocytes TAG content. In the liver, DGAT has a role in synthesizing TAG from either fatty acids synthesized *de novo* or from fatty acids taken up from the circulation. Two DGAT enzymes were identified, DGAT1 and DGAT2. While DGAT1 seems to be associated to TAG synthesis and VLDL secretion stimulation [15], DGAT2 appears to catalyze the final step in the pathway leading to *de novo* synthesis and intracellular accumulation of TAG [16]. SREBPs are major transcription factors that regulate the expression of genes involved in lipid synthesis in the liver. Three isoforms exist, SREBP-1a, −1c and −2 [17, 18]. SREBP-2 activates genes involved in cholesterol metabolism, whereas SREBP-1 regulates genes involved in the metabolism of fatty acids [240]. SREBP-1c is the predominant isoform expressed in liver [18] and is mostly responsible for the expression of genes involved in hepatic fatty acid biosynthesis [19].

Over the last decade, interactions of PCB126 with nervous [20] and reproductive [21] metabolic activities for instance, have attracted much interest. However, the effects of PCB126 on the liver, specifically on the underlying molecular mechanisms leading to NAFLD have not yet been defined. Therefore the purpose of this research study is to understand the molecular effects of PCB126 exposure on VLDL production, estimated through MTP, and on hepatic lipid synthesis, via DGAT2 and SREBP1c using human cell culture and animal model.

## Methods

### Cell culture

PCB126 was obtained from Ultra Scientific (North Kingstown, RI, USA) and dimethyl sulfoxide (DMSO) was obtained from Sigma-Aldrich (St. Louis, MO, USA). PCB126 was dissolved in DMSO as stock solution and diluted with Differentiation Medium (Zenbio Inc, North Carolina, USA, cat# DM-2) to specific concentration before being added to the cells in culture. The final concentration of DMSO was never greater than 0.3 % (v/v). Human hepatocytes (ZenBio Inc., Durham, NC) were initially plated at a density of 9.4 10^4^ cells/well. Following a 6 to 8 h incubation period for cell attachment, hepatocytes were exposed to PCB126 [2.5uM] for 72 h [22]. Control group was treated in DMSO (0.3 %) and chloroquine [25uM] served as a positive control. Cells were incubated at 37 °C in a humidified 95 % air-5 % CO_2_ atmosphere incubator in 48 well plates.

Lipid quantification was assessed in human hepatocytes using steatosis colorimetric assay (Cayman Chemical Company, Ann Arbor, Michigan).

### Animal care

Female Sprague–Dawley strain rats (Charles River, St-Constant, Quebec, Canada), weighing 200-250 g upon their arrival were housed in pair and had ad libitum access to food and tap water. Their environment was controlled in terms of light (12:12-h light–dark cycle starting at 6:00 AM), humidity and room temperature (20-23 °C). All rats were fed a standard diet (Harlan 2018 Rodent Diet, Harlan Teklad Laboratory) and had access to water ad libitum. The sham group received a single ip injection of corn oil (0.14 ml/kg) while experiment group received PCB126 (1.05 umol/kg) dissolved in corn oil [23]. All procedures were approved by the Animal Care Committee of the University of Ottawa and adhered to the guidelines established by the Canadian Council on Animal Care.

### Tissue sampling

Following 7 days after the injection, and after complete anaesthesia (sodium pentobarbital, 65 mg/kg, ip.), the abdominal cavity was rapidly opened following the median line of the abdomen. Blood was rapidly (<45 s) drawn from the abdominal vena cava (~4 ml) and transferred in tubes pretreated with EDTA (15 %). Blood was centrifuged (3 000 g for 10 min, 4 °C) and the plasma were stored at − 80 °C until analyses. The liver was excised, the median lobe immediately frozen used for triacylglycerol determination and protein quantification.

### Western immunoblotting

Briefly, 200 mg of liver was homogenized in Lysis Buffer (extraction buffer) with Na3VO4 1 mM and protease inhibitor cocktail using a polytron, sonicated and centrifuged at 13,000 g, 4 °C for 20 min. The infranatant was collected with a blunt-tipped Pasteur pipette and stored at -80 °C until protein determination. All samples (50 μg of proteins) were separated on a 10 % SDS-polyacrylamide gel and electrotransferred onto nitro-cellulose membranes. Membranes were blocked 1-h in Phosphate-buffered saline containing 0.05 % Tween 20 (PBST 0.05 %) and 5 % non-fat dry milk at 4 °C. The blot was then incubated with primary antibody overnight. After five washes in PBST (0.05 %), the membrane was incubated for 45 min with secondary antibody (Novus Biologicals, Littleton, CO) at room temperature. Then the membrane was washed five times for 15 min each time in PBST (0.05 %) before revealing with West Pico (Fisher Scientific Company). The resulting signal was acquired with ChemiDoc-It Imaging System (Fisher Scientific Company) and the bands were quantified with the use VisionWorks®LS (UVP, LLC, Upland, CA) software and expressed as arbitrary units. Equal protein loading was determined using ß-actin (Sigma, Saint Louis, Missouri, USA).

### Analytical procedures

Plasma TAG levels were determined with an enzymatic colorimetric assay available from SIGMA (Saint Louis, Missouri, USA). Liver TAG concentrations were estimated from glycerol released after ethanolic KOH hydrolysis by using commercial kits from SIGMA (Saint Louis, Missouri, USA).

### Statistical analysis

Values are expressed as mean ± S.E. Statistical analyses were performed using a one-way analysis of variance (ANOVA) for non-repeated measures. Fisher’s LSD post hoc test was used in the event of a significant (*p* < 0.05) F ratio. Significance was accepted at *p* < 0.05. All analyses were performed using SPSS Statistics 20.0, SPSS Inc., Illinois, USA.

## Results

### Cells

**Fig. 1:**
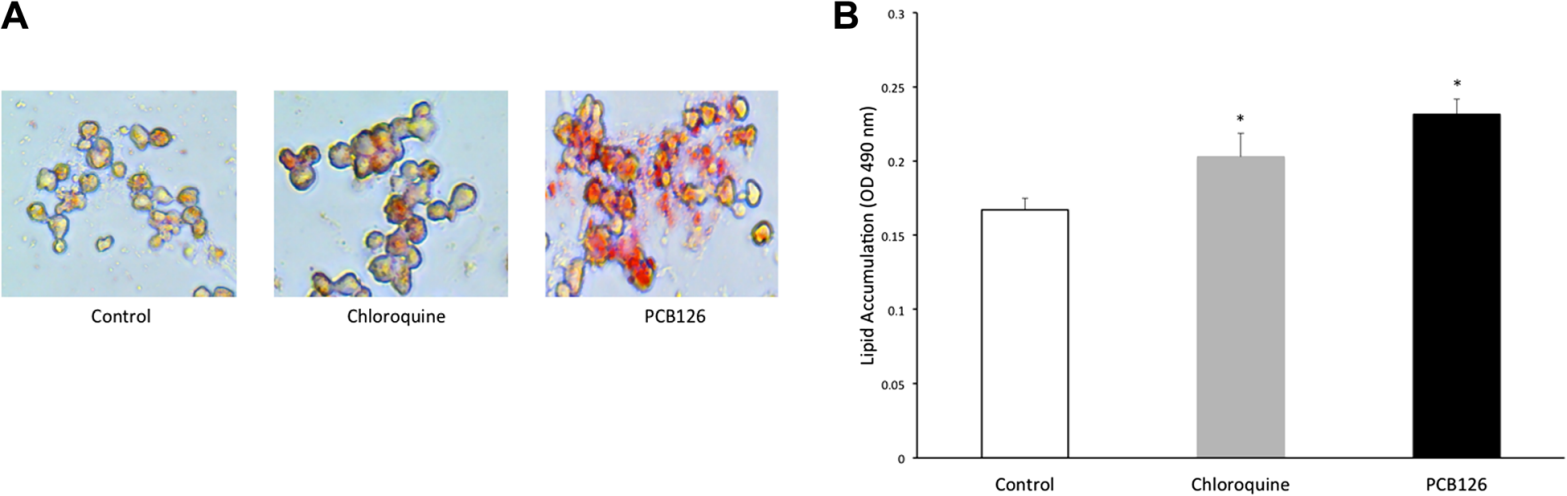
Lipid accumulation in hepatocytes. (**a**) Neutral lipid staining in hepatocytes using Oil-Red-O exposed to DMSO (Control), Chloroquine and PCB126 [2.5 μM] viewed by light microscopy (magnification 40x). (**b**) Lipid quantification by colorimetry after dye extraction from lipid droplet (OD 490 nm). *Significantly different from Control rats *p* < 0.05. *n* = 8-11. Control = White; Chloroquine = Grey; PCB126 = Black

As illustrated in Fig. 1a hepatocytes lipid accumulation seen by microscope by Oil-Red-O and (B) measured by colorimetry (OD 490 nm) after Oil-Red-O extraction from lipid droplets, was 38 % greater in cells treated with PCB126 [2.5uM] for 72 h (0.23 ± 0.01 OD_490nm_) compared to Control (DMSO) (0.17 ± 0.007 OD_490nm_) group (*P* < 0.001). Although PCB126 treated cells depicted 14 % higher lipid content than Chloroquine, a potential lipid droplet infiltration inducer (0.20 ± 0.01 OD_490nm_), statistical significance was not reached (*P* < 0.08). The cells treated with Chloroquine depicted 21 % higher lipid content than the Control group (*P* < 0.04).

### Rats

**Fig. 2:**
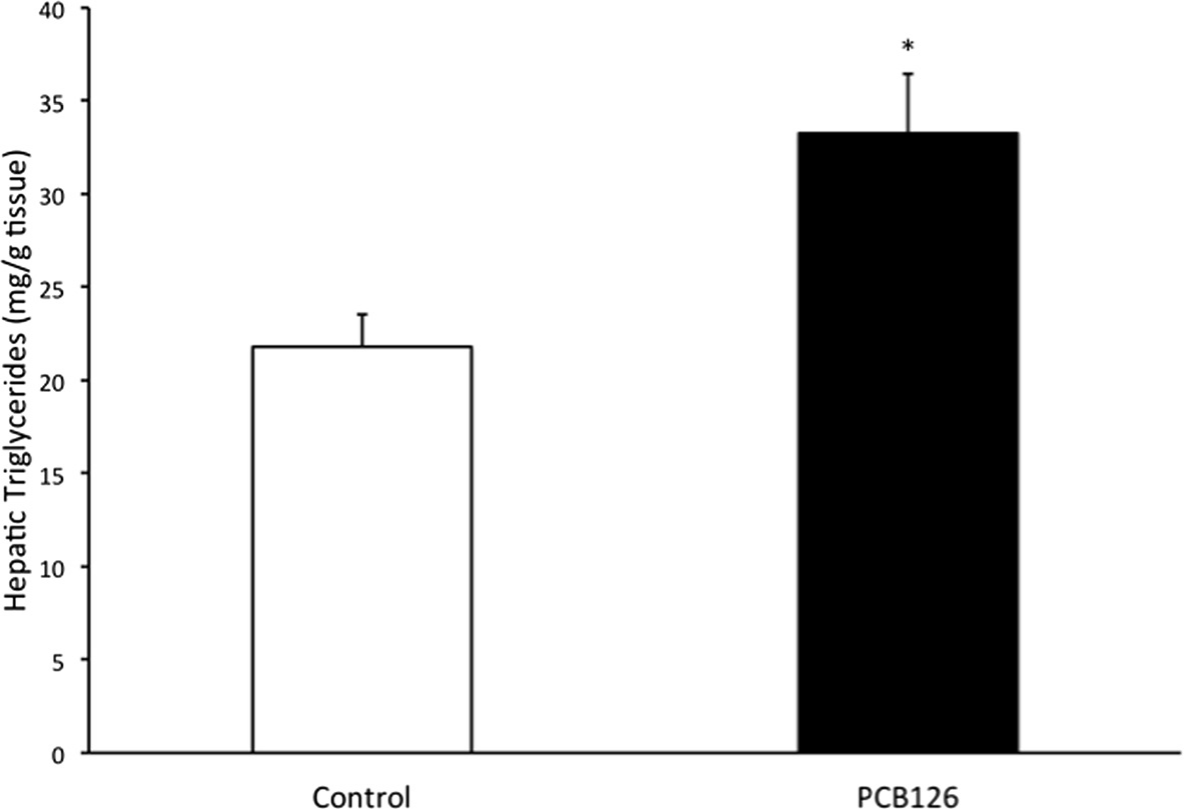
Hepatic TAG content (mg/g). *Significantly different from Control *p* < 0.001. *n* = 9-11. Control = White; PCB126 = Black

**Fig. 3:**
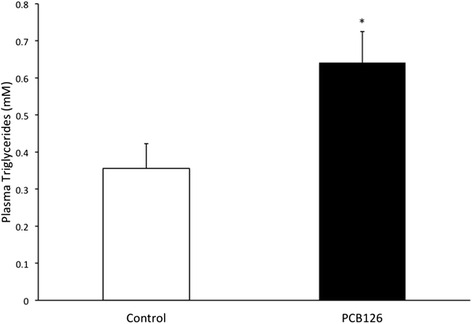
Plasma Triglycerides concentrations (nM). *n* = 7. *Significantly different from Control group *p* < 0.02. Control = White; PCB126 = Black

**Fig. 4:**
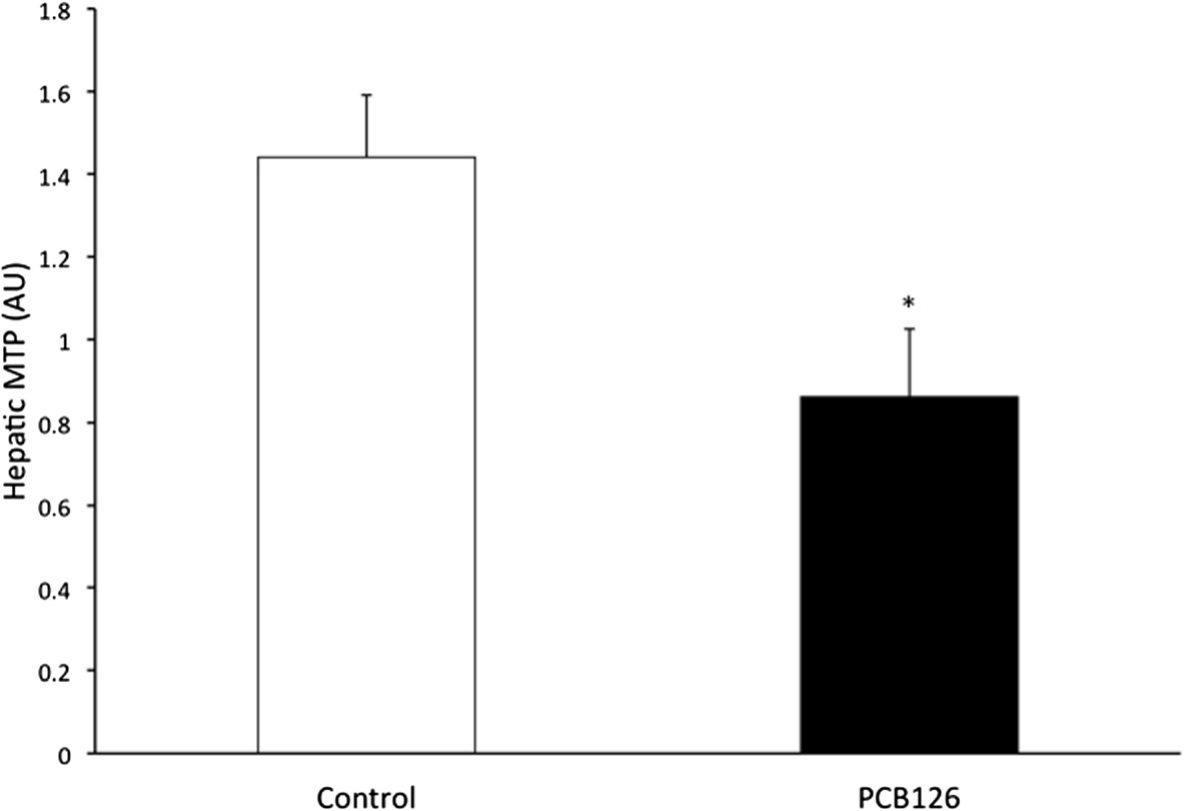
MTP. Western blotting of MTP measured by optical density (OD). *n* = 8. *Significantly different from Control rats *p* < 0.02. Control = White; PCB126 = Black

**Fig. 5:**
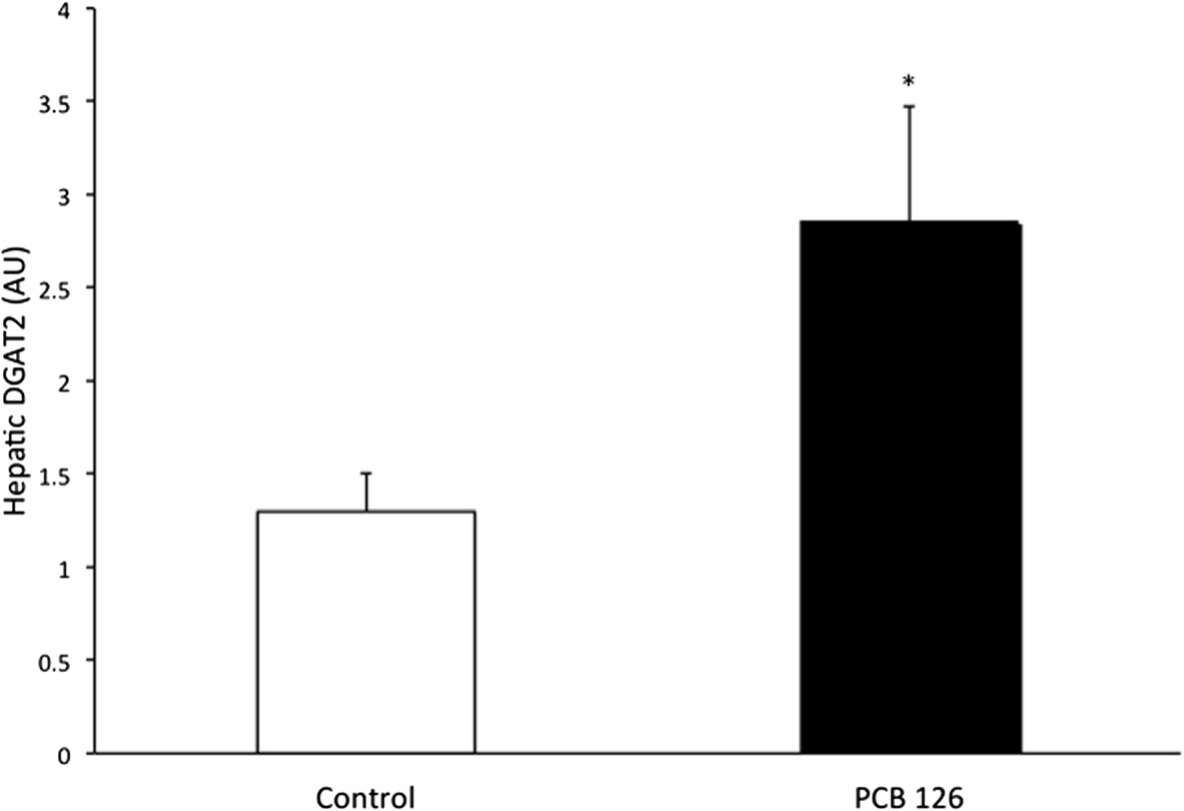
DGAT2. DGAT2 was quantified by optical density (OD) using Western Blot. *n* = 5-7. *Significantly different from Control group *p* < 0.02. Control = White; PCB126 = Black

**Fig. 6:**
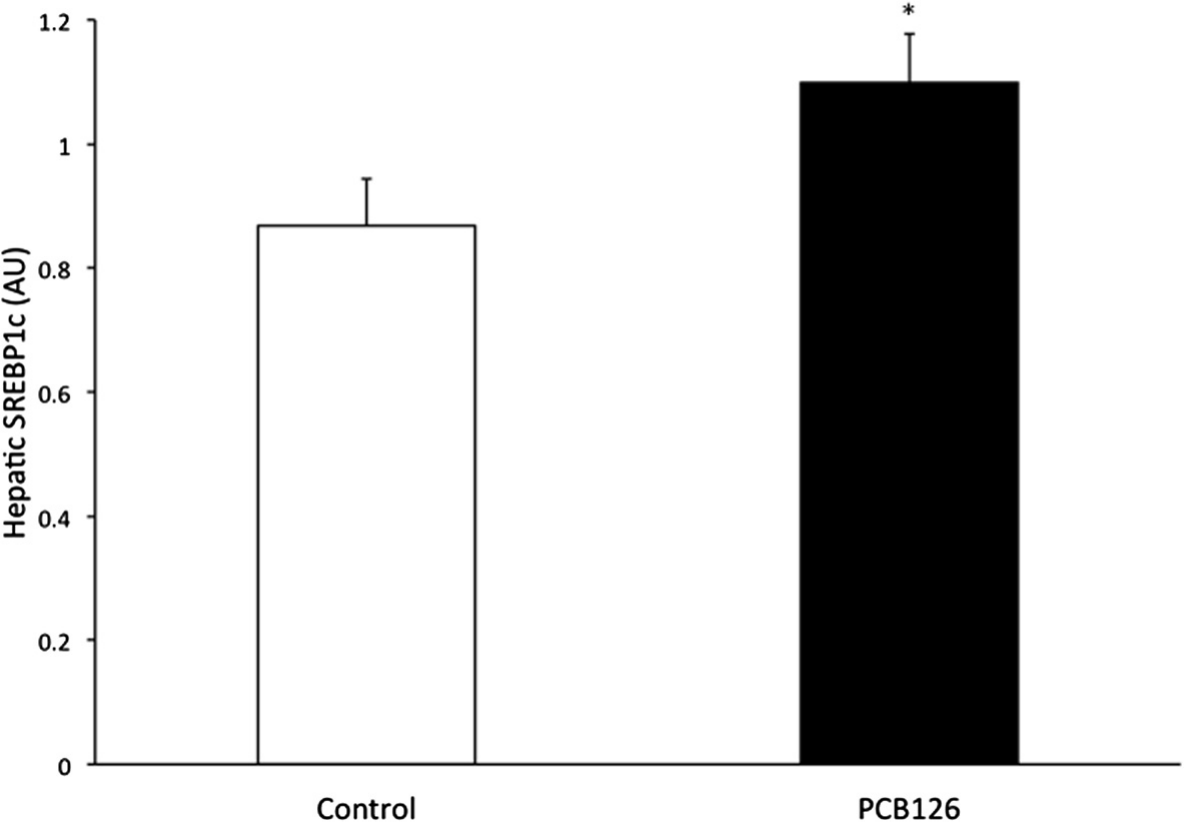
SREBP1c. SREBP1C protein content measured by Western Blot and reported by optical density (OD). *n* = 8. *Significantly different from Control rats *p* < 0.05. Control = White; PCB126 = Black

PCB126 injections in rats resulted in higher hepatic TAG content (64 %) compared to Control group injected with Corn oil (21.3 ± 1.3 vs 35.2 ± 3.1 mg/g; *P* < 0.001) (Fig. 2). Rats treated with PCB126 had 80 % more plasma TAG compared to Corn Oil group (0.35 ± 0.06 vs 0.64 ± 0.08 mM, *P* < 0.02) (Fig. 3). All hepatic protein contents are expressed in arbitrary units (AU). Immunoblotting of MTP resulted in a 40 % decrease in PCB126-injected rats compared to Corn Oil group (1.4 ± 0.1 vs 0.8 ± 0.1 AU; *P* < 0.02) (Fig. 4). In contrast, protein content of DGAT-2 revealed to be 120 % higher (1.29 ± 0.2 vs 2.85 ± 0.6 AU; *P* < 0.02) (Fig. 5) and SREBP1c was 26.5 % greater (0.87 ± 0.07 vs 1.09 ± 0.07 AU; *P* < 0.05) (Fig. 6) compared to control rats.

## Discussion

In the present study, we observed that seven days of exposure to PCB126 lead to significant increases of lipid content in hepatocytes and in liver of rats. Our results strongly suggest that the presence of PCB126 is related to important hepatic fat accumulation. Accordingly, Wahlang et al. (2013) revealed that PCB 153 exposure in high fat-fed mice was associated with an increased hepatic TAG content in C57Bl/6 J mice compared to vehicle control group [24]. While, PCB-77 induced obesity in C57B/6 mice and promoted differentiation of 3T3L1 adipocytes [25]. Together, these results imply an important association between PCBs exposure and increased fat accretion.

There are likely multiple factors involved in hepatic fat accumulation. We report an important decrease in MTP protein content in PCB126 treated rats compared to Corn oil injected group (Fig. 4). Deficiency of MTP gene in mice [26, 27] and gene polymorphism in humans [28] have been associated with the development of NAFLD. Also, pharmacological inhibition of MTP in rats and humans increased liver TAG content ranging from 30–1000 % compared to control counterparts [29, 30]. Together, these results suggest that variations of the expression and activity of MTP significantly affect hepatic lipid. However, to our knowledge there are no published studies on the effects of PCBs on hepatic MTP content and liver TAG accumulation. Therefore in our study, we can conclude that an altered expression of MTP resulting from PCB126 exposure might potentially cause important TAG accumulation in hepatocytes.

MTP plays a crucial role in the synthesis and hepatic VLDL secretion, thus on plasma TAG levels. Pharmacological inhibition of MTP has been mainly used in the treatment of hypertriglyceridemia in humans (Cuchel 2013; Raval 2012). Administration of a MTP inhibitor for two weeks resulted in a 600 % increase in concentrations of TAG liver and a decrease of 100 % of plasma TAG in comparison to control rats [31]. In mice, deletion or deficiency of hepatic MTP gene results in alteration of the VLDL secretion, reflected by significantly lower plasma concentrations of plasma triglycerides levels compared to control mice [26, 27, 32]. Conversely, an increased MTP protein content (161 %) in the liver results in lipoprotein overproduction (30 %) in a fructose-fed hamster model of insulin resistance [33–35]. Although, our results reveal that despite the fact that MTP content was lower by 40 %, PCB126 injected rats in our study showed nearly 80 % higher plasma TAG concentrations than in control group (Fig. 3). These results are in line with several studies that have reported positive associations between PCBs and serum lipid in humans [36] and animals [37–39]. Mice had significant increase (269 %) in plasma TAG concentrations following oral administration of Aroclor 1254 for 14 days when compared with the control group [39]. As well, continuous orally daily exposure of various concentrations of Aroclor 1254 monkeys for 152 weeks in female rhesus was associated with increased in plasma TAG ranging from 30–56 % [37]. Whereas MTP content was lower in our PCB126 exposed rats, we hypothesize that increases in plasma TAG levels may occur due to increased hepatic synthesis of TAG and production of larger VLDL particles. Production of VLDL with a larger size implies that fewer particles are being secreted to account for the similar TG production rates. In support of our hypothesis, one study reported a 30 % increase in size of VLDL particles measured directly in the ER in male rats injected with PCBs [40]. Furthermore, in normal and T2D subjects, an overproduction of large VLDL particles is the major determinant of plasma TG levels and is mainly caused by liver fat content [41, 42].

Hepatic TAG content is partly regulated by fatty acid synthesis and esterification processes. DGAT2 catalyzes the final step in the G3P pathway leading to TAG synthesis. Our results revealed a substantial increase of 120 % in hepatic DGAT2 protein content in our PCB126 treated rats compared to Corn oil group (Fig. 5). In line with our results, a recent study reported that DGAT-2 gene was differentially expressed (4.8-fold) in mouse lungs after 3-weeks of polluted ambient air exposure relative to control mouse [43]. Mice overexpressing hepatic DGAT2 showed a 260 % increase in liver TAG concentrations [44]. While suppression of DGAT2 with antisense oligonucleotide treatment improves hepatic steatosis in a diet induced NAFLD [45] and obesity [46, 47] rat models. On the whole, these results suggest an important contributing role of DGAT2 in lipid accumulation and possibly leading to the development of NAFLD in our rats contaminated with PCB126.

Moreover, a recent study has reported that DGAT2 is specialized for glycerol 3-phosphate (G3P) incorporation into TAG, however it seems that the origin of these G3P are not from exogenous preformed fatty acid [48]. A possibility emerged that DGAT2 may be specialized for the utilization of *de novo* synthesized fatty acids [49]. Endogenously fatty acid synthesis occurs predominantly through *de novo* lipogenesis (DNL), both liver and adipose tissue. SREBP1c has been closely related to development of hepatic steatosis [50, 51] and hypertriglyceridemia [50]. Our results shows that our steatotic rats exposed to PCB126 depicted significantly higher SREBP1c protein concentration in their livers (Fig. 6). Similarly, a significant increase in the expression level of SREBP1c was induced in rats fed farm salmon containing various POPs [52].

## Conclusions

Environmental pollutants have emerged as new contributing actors for their suspected endocrine and metabolic disruption activity leading to the development of NAFLD. The results emerging from this study bring valuable molecular information on how lipotoxic compounds such as PCB126 interfere with the normal functioning of vital metabolic pathways in the liver and are clearly associated with the molecular development of NAFLD.

A potential weakness of this study includes the i.p. route of administration of PCB126 and that a single dose of PCB 126 was used. PCBs are known to concentrate within adipose tissue and liver. Although PCB 126 levels were not measured in our livers, we expect them to be in line with to those in the National Toxicology Program Report [53]. Further *in vivo* studies are needed to more fully understand the mechanisms involved in PCB126 induced NAFLD. It already been reported that alteration in oxidative pathways may lead to ectopic fat accumulation in various organs including liver [54, 55]. Also, simultaneous co-exposures to chemicals and whether they may act in an additive, synergistic, or antagonistic manner must be taking in account in future study designs.
